# Norovirus: a novel etiologic agent in hemolytic uremic syndrome in an infant

**DOI:** 10.1186/s12882-019-1427-6

**Published:** 2019-07-05

**Authors:** Ghadi Abu Daher, Bilal Aoun, Fatima Jaafar, Sarah Khafaja, Sami Sanjad

**Affiliations:** Department of Pediatrics and Adolescent Medicine, American University of Beirut Medical Center, P.O.Box 11-0236/E29, Beirut, Lebanon

**Keywords:** Hemolytic uremic syndrome, Norovirus, Acute kidney injury, Peritoneal dialysis, Complement pathway

## Abstract

**Background:**

Hemolytic uremic syndrome is a rare thrombotic microangiopathy usually seen in infants and children below the age of 5 years. It usually follows a bout of bloody diarrhea caused by Shiga toxin producing E coli and is characterized by microangiopathic hemolytic anemia, thrombocytopenia and acute kidney injury. We report the first case of hemolytic uremic syndrome in an infant following Norovirus gastroenteritis.

**Case presentation:**

A nine-month-old male infant, was admitted with an 8-day history of watery, non-bloody diarrhea, vomiting and decreased oral intake. Physical exam revealed normal blood pressure, pallor and generalized edema. Laboratory findings were significant for microangiopathic hemolytic anemia, thrombocytopenia and azotemia. Stool studies with Multiplex Qualitative reverse transcriptase PCR were positive for Norovirus GI/G II. His clinical course was unusually severe, complicated by oligoanuria and worsening uremia requiring peritoneal dialysis but with eventual complete recovery.

**Conclusions:**

To our knowledge this is the first case of Norovirus associated HUS in an infant. Given the ubiquity of this virus as a major cause of diarrhea, together with the increased availability of Multiplex Qualitative PCR in reference laboratories, it is quite possible that we shall be seeing more such cases in the future.

## Background

Hemolytic uremic syndrome (HUS) is a rare thrombotic microangiopathy (TMA) with a mean annual incidence of 6.1 cases/million children less than 5 years of age and an overall case fatality rate of 4% [1]. The primary damage to the vascular endothelium is responsible for the triad of microangiopathic hemolytic anemia, thrombocytopenia and acute kidney injury (AKI). Pediatric HUS is classified either as: 1) typical and caused by Shiga toxin producing E.coli (STEC) and accounts for 90% of cases or 2) atypical, subdivided into familial HUS or non-familial HUS which are usually secondary to inherited or acquired abnormalities in the alternate complement pathway [2]. Patients with STEC HUS develop acute kidney injury typically 2–12 days after the onset of bloody diarrhea [3]. Treatment is supportive, blood transfusion if deemed necessary and temporary dialysis in case of severe acute kidney injury. Fortunately the mortality rate for STEC-HUS has decreased to less than 5% with a 70% complete recovery after an episode acute kidney injury [4].

We report an unusual case of HUS with severe anuric AKI in association with Norovirus (previously Norwalk virus) induced gastroenteritis in a nine-month-old baby which, to our knowledge, is the first infant where such a causal association is documented. We also present a brief review of other viruses reported to cause HUS and TTP.

## Case presentation

A nine-month-old male infant, was admitted with an 8-day history of watery, non-bloody diarrhea, vomiting and decreased oral intake. The baby was previously healthy and had a negative medical history.

On physical examination, the patient was pale and irritable with generalized body edema, tachypnea (rate 36/min) and tachycardia (HR140/min) but no rales or murmurs were heard. No other abnormalities were noted.

Initial investigations revealed leukocytosis (19 × 10^3^), anemia (hemoglobin 7.7 g/L, hematocrit 22%) and thrombocytopenia (platelets 62× 10^3^). Serum creatinine was 2.5, BUN, 57 uric acid, 7.6 mg/dl respectively, LDH 2293 IU/L. (reference value 265 I U/L). Peripheral blood smear revealed evidence of microangiopathic hemolysis with schistocytes and helmet cells. Abdominal ultrasound showed echogenic but normal sized kidneys. The patient was admitted to the pediatric intensive care with the diagnosis of hemolytic uremic syndrome for possible dialysis.

During his stay, the patient had persistent diarrhea, decreased oral intake, oligoanuria and generalized body edema and hypertension. There was no response to high doses of intravenous furosemide (urine output less than 0.5 ml/kg/hour). Because of progressive deterioration in kidney function (creatinine reaching 5.2 mg/dl, blood urea nitrogen 88 mg/dl), persistent oligoanuria and worsening microangiopathic hemolysis and thrombocytopenia (hemoglobin of 5.4 g/dl and platelets of 23× 10^3^) peritoneal dialysis was started after 48 h of hospitalization. The patient also received one unit of packed RBC transfusion.

Stool studies with Multiplex Qualitative reverse transcriptase PCR were negative for *Salmonella, Shigella, Campylobacter*, *Yersinia*, enterohemorrhagic *E coli*; enteropathogenic *E coli* (EPEC), enterotoxigenic *E coli* (ETEC), enteroinvasive *E coli* (EIEC) and Shiga-like toxin-producing E.coli (STEC) stx1/stx2, Rotavirus A, Adenovirus, Astrovirus, but were positive for Norovirus GI/G II.

Additional relevant laboratory studies included massive albuminuria with an Ualb/Cr 97 mg/mg (ref value < 0.2), low C3 and C4 complement of 0.56 g/L (ref value 0.9–1.8 g/L) and 0.07 g/L (reference value 0.1–0.4 g/L) respectively.

**Fig. 1:**
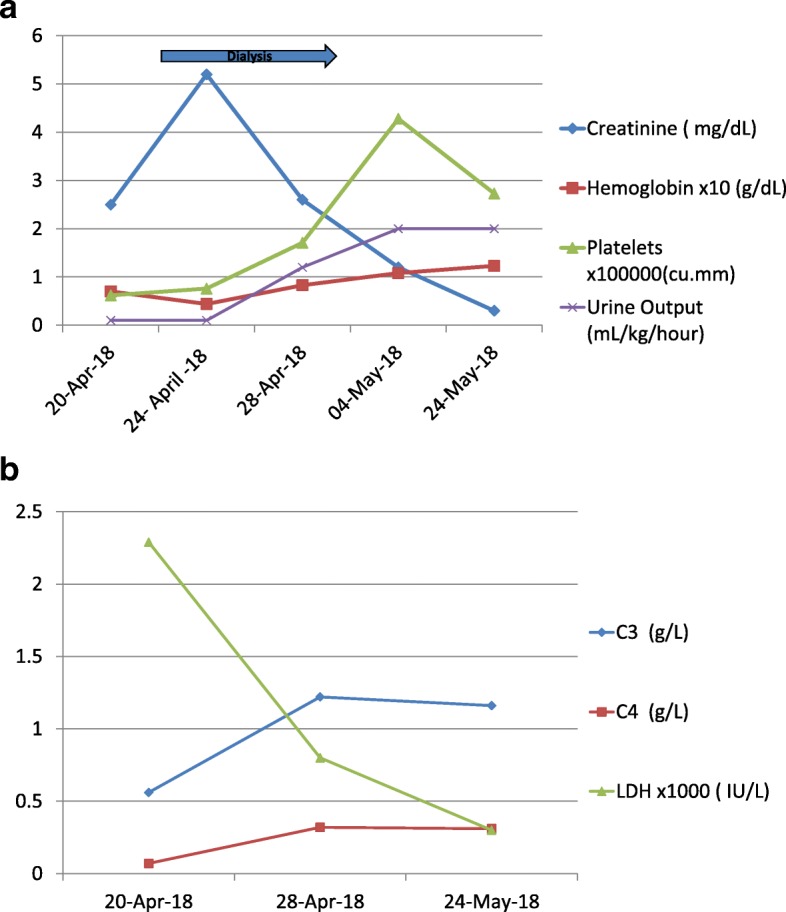
**a** Urine output and select laboratory markers. **b** Changes in complement and LDH levels. Figure 1a Renal functional parameters and hematological changes. Figure 1b Complement, LDH and Albuminuria during hospitalization

After 48 h of continuous peritoneal dialysis, the patient improved clinically with progressive decrease in edema and gradual increase in urine output. Peritoneal dialysis was continued for 5 more days and his kidney function improved steadily and normalized by the 10th day when his serum creatinine was 0.3 mg/dl, and albuminuria was down to 0.13 mg/mg creatinine. Three weeks after the onset of the disease, C3 and C4 had returned to normal levels at 1.16 g/L and 0.31 g/L respectively. The patient’s hospital course and laboratory data are depicted in Figs. 1a and b. The patient has been followed for 11 months since the onset of his illness and shows no evidence of residual damage with normal renal, hematologic and complement profile. He is normotensive and has no microalbuminuria.

## Discussion and conclusions

The overall incidence of diarrhea-associated HUS is rare at around 2.1 cases per 100,000 persons/yr., with a peak incidence of 6.1 per 100,000/yr. in children younger than 5 years. While rare in occurrence, HUS is a very common cause of AKI in children, particularly in infants below the age of 4 years [2]. The vast majority of cases occur following a bout of bloody diarrhea caused by STEC, usually O157: H7 serotype enterohemorrhagic *E. coli.* The disease is characterized by microvascular thrombosis, primarily in the renal vasculature, but occasionally in multiple organs, microangiopathic hemolytic anemia and thrombocytopenia. Acute kidney injury may be mild with transient elevation of urea and creatinine and oliguria but in more than 50% it is severe and protracted, necessitating renal replacement therapy in the form of hemodialysis or peritoneal dialysis [3] as was the case with our patient. Although the mortality in STEC-HUS in children has decreased steadily over the years and is now at less than 5%, as many as 25% of survivors may be left with residual renal impairment including proteinuria, hypertension and reduced renal function [5].

While the vast majority of cases of diarrhea-associated HUS are secondary to STEC (O157:H7), a number of viruses have been shown recently to play an important role in the pathogenesis of thrombotic microangiopathies. Both DNA and RNA viruses have been implicated, with the cytomegalovirus (CMV) playing an important role in the development of TMA in the former, particularly in immunocompromised patients and in association with renal transplantation. Other DNA viruses (varicella, EBV, adenovirus, parvovirus) related TMAs are limited to individual case reports [6].

Of the RNA viruses, the human immunodeficiency virus (HIV) is the most common in causing TMA. Both thrombotic thrombocytopenic purpura (TTP) and HUS have been reported in HIV patients and in patients with AIDS with a higher morbidity and mortality in the latter group [7]. Direct endothelial injury and secondary ADAMTS13 deficiency were likely responsible for the pathogenesis of TMA in these patients. Other RNA viruses associated with HUS and/or TTP include the coxsackie and echoviruses as well as the Influenza virus (H1N1) with several cases reported in children and adolescents [8]. Single case reports of HUS have been published for hepatitis A and rotavirus [6], and few for norovirus in adults [9, 10]. Cases of norovirus induced HUS are still rare in pediatric population but have been documented in 2 older children, a 9 year-old female and a 7-year-old male respectively. The latter had a concomitant Clostridium detected in the stool specimen [11, 12].

Noroviruses constitute a major cause of foodborne illness and are responsible for at least 50% of all gastroenteritis outbreaks worldwide [13]. They are divided into at least six geno groups, designated GI-GVl, based on the amino acid sequence in the major structural protein [14] and further subdivided into at least 38 genotypes. Norovirus gastroenteritis is characterized by sudden onset of fever, nausea, vomiting, watery diarrhea and abdominal cramps with an incubation period of 12 to 48-h. Symptoms typically last 24 to 60 h. Reverse-transcriptase PCR or enzyme immunoassay testing of stool specimens can confirm the diagnosis. Treatment is usually symptomatic and supportive, limited to oral or intravenous hydration [15].

Two adults with suspected norovirus related HUS have been reported. The first was an 82-year Japanese man with hypertensive nephrosclerosis. The patient developed AKI following a bout of non-bloody diarrhea superimposed on his CKD stage 3. There was evidence of TMA and he required acute and subsequently, chronic hemodialysis. Norovirus antigen was identified in a fecal sample by enzyme immunoassay [9]. The second was a 42-year old female with chronic interstitial nephritis, who presented with worsening renal function and markers of nonimmune vascular hemolysis two years post renal transplant, and was found to have norovirus by enzyme immunoassay on fecal sample [10]. In our patient with HUS and severe AKI, Norovirus Gl/Gll was detected in 2 separate stool specimens by multiplex reverse transcriptase PCR, while all other gastrointestinal pathogens, including STEC, stx1/stx 2 were negative. This provides substantive evidence for the role of Norovirus in the causation of HUS. Detection of stool pathogens by multiplex PCR has been found to be a reliable method and has become commercially available [16]. We believe that the virus or viral antigens caused endothelial damage by direct invasion initiating an inflammatory response and activation of the complement cascade with platelet activation and aggregation with subsequent microangiopathic hemolysis. Unlike atypical HUS and some cases of STEC HUS where the alternate complement pathway is activated and only C3 is low [17], our patient’s low C3 and C4 suggests activation of the classical complement pathway as is usually seen in post infectious glomerulonephritis, lupus nephritis and other immune complex mediated renal diseases. While beyond the scope of this report, the main difference between these two pathways is that the C3 convertase in the alternate pathway is C3bBb and activation occurs spontaneously and continuously, whereas in the classical pathway, the convertase is C4bC2b with activation occurring via C1qrs complex and cleavage of C4b and C2b. That may explain why the early components of the complement cascade (C1-C4) are low with classical pathway activation whereas a low C3 alone is suggestive of alternate pathway activation [17, 18]. We realize that since we did not look for mutations or antibodies to complement proteins (CFH, CFI, and MCP), atypical HUS, with the Norovirus infection acting as a trigger in a susceptible individual, cannot be ruled out and this is a limitation in our report. However, the complete clinical recovery, together with the return to normal of both complement components (C3, C4) without the need of plasma therapy or Eculizumab makes that possibility less likely. Also, the patient has been followed up for 11 months since hospital discharge with serial measurements of his blood pressure, hemogram, kidney function, complement levels and urine albumin excretion which have all been normal. Long term follow up is needed, however, and in the event of a relapse, genetic studies to detect mutations in complement proteins will be performed.

In conclusion, we have presented a case of severe HUS with anuric AKI in association with a Norovirus-related gastroenteritis. To our knowledge this is the first time such a causal relationship has been documented in an infant. With the routine immunization against rotavirus in infants, the prevalence of Norovirus gastroenteritis and its detection in stool samples by Multiplex PCR in this population is likely to rise, thus more cases of Norovirus-induced HUS may be diagnosed in the future, particularly in STEC negative patients with HUS.

## Data Availability

Not applicable.

## Abbreviations

AKI: Acute kidney injury
HUS: Hemolytic Uremic Syndrome
LDH: Lactate dehydrogenase
STEC: Shiga toxin producing E coli
TMA: Thrombotic microangiopathy
TTP: Thrombotic thrombocytopenic purpura
